# Epigenetic Targets in Schizophrenia Development and Therapy

**DOI:** 10.3390/brainsci13030426

**Published:** 2023-03-01

**Authors:** Agnieszka Wawrzczak-Bargieła, Wiktor Bilecki, Marzena Maćkowiak

**Affiliations:** Department of Pharmacology, Laboratory of Pharmacology and Brain Biostructure, Maj Institute of Pharmacology, Polish Academy of Sciences, Smętna 12, 31-343 Kraków, Poland

**Keywords:** antipsychotics, genetics, neurodevelopment, environment, psychosis, pharmacotherapy, animal models, human studies, adolescence, treatment-resistance

## Abstract

Schizophrenia is regarded as a neurodevelopmental disorder with its course progressing throughout life. However, the aetiology and development of schizophrenia are still under investigation. Several data suggest that the dysfunction of epigenetic mechanisms is known to be involved in the pathomechanism of this mental disorder. The present article revised the epigenetic background of schizophrenia based on the data available in online databases (PubMed, Scopus). This paper focused on the role of epigenetic regulation, such as DNA methylation, histone modifications, and interference of non-coding RNAs, in schizophrenia development. The article also reviewed the available data related to epigenetic regulation that may modify the severity of the disease as a possible target for schizophrenia pharmacotherapy. Moreover, the effects of antipsychotics on epigenetic malfunction in schizophrenia are discussed based on preclinical and clinical results. The obtainable data suggest alterations of epigenetic regulation in schizophrenia. Moreover, they also showed the important role of epigenetic modifications in antipsychotic action. There is a need for more data to establish the role of epigenetic mechanisms in schizophrenia therapy. It would be of special interest to find and develop new targets for schizophrenia therapy because patients with schizophrenia could show little or no response to current pharmacotherapy and have treatment-resistant schizophrenia.

## 1. Introduction

Schizophrenia is a chronic psychiatric condition that affects a person’s thinking, feeling, and behaviour. Schizophrenia is a syndrome with symptoms defined by signs of psychosis, which affect a person’s emotional responsiveness and social interactions. The exact causes of schizophrenia are unknown, however, a combination of physical, genetic, psychological, and environmental factors was suggested as factors that make a person prone to schizophrenia, with stressful events triggering a psychotic episode in some instances. Signs and symptoms may vary, but usually involve delusions, hallucinations, disorganized speech, as well as grossly disorganized behaviour, and an impaired ability to function [1]. These symptoms are usually divided into three groups: positive, negative, and cognitive dysfunction (executive function) [2]. Positive symptoms, affecting the individual’s thoughts or behaviours include delusions, hallucinations, inexplicable behavioural changes, and thought disorder. Negative symptoms, regarded as a core component of schizophrenia, are defined as a reduction in normal functions either related to motivation and interest (e.g., avolition, anhedonia, and asociality) or expressive functions (e.g., blunted affect, and alogia) [3]. Cognitive deficits, which, on average, are around two standard deviations below that in healthy controls, appear to be distinct from the positive and negative symptoms, and poor learning and retention of verbal information are some of the most consistent findings across research studies [4]. Although primarily considered a disorder of the central nervous system, autonomic dysfunction, in the form of increased sympathetic activity and decreased parasympathetic activity, is postulated to be implicated in schizophrenia and its treatment [5]. A constellation of metabolic aberrations that constitute metabolic syndrome, including obesity, dyslipidemia, insulin resistance and diabetes, and hypertension, contribute to a reduced life expectancy compared to healthy people [6].

The first symptoms of schizophrenia begin in late adolescence or young adulthood. Evidence indicates that 50% of cases had an acute onset, and 50% had a long prodrome of the disease [7,8]. The study of the prodrome suggests the beginning of negative symptoms tends to occur about five years before the initial psychotic episode, and the onset of positive symptoms appears closer to the first hospitalization [9,10]. 

The start of schizophrenia also depends on sex, and in men, the first sign is observed in their late teens to early 20s, and in women in their late 20s to early 30s. Available data suggest that men have a 30–40% higher lifetime risk of developing schizophrenia than women [10], although several reviews did not find sex differences in the prevalence of the illness. This discrepancy between studies may reflect the more severe clinical picture for men and that women often get diagnosed later in life [11].

Several hypotheses were implicated in the pathophysiology of schizophrenia, particularly involving dopamine, glutamate, and γ-aminobutyric acid (GABA). The dopaminergic hypothesis of schizophrenia proposes that positive symptoms result from hyperactivity of dopaminergic neurotransmission in limbic pathways, while negative symptoms are thought to arise from hypodopaminergic functioning in the frontal structures. Data from recent studies are consistent with the hypothesis that schizophrenia is associated with a hypodopaminergic state in the cortex and may be linked to a hyperdopaminergic state in the striatum [12,13]. The current diagnostic criteria are based on subjective evidence; however, clinical characteristics alone are of limited predictive value, especially during the early stage of the disease. Therefore, biological predictors are urgently needed in the clinical diagnosis of schizophrenia. Biomarkers of schizophrenia may be divided into peripheral (blood-based biomarkers are useful tools to reveal some processes in the brain) or central biomarkers and classified into persistent markers, such as genetic markers to identify the risk of schizophrenia to benefit early diagnosis, as well symptom-related markers to evaluate the progress of the disorder and the effectiveness of the therapy. One of the most recognised genetic markers is a deletion at 22q11. 2, which has been estimated to affect one in four schizophrenia cases and induce a 30% lifetime risk of schizophrenia [14]. The neuroinflammatory idea has linked microglia activation to cognitive decline, while brain energy theory suggests that energy metabolism is compromised in schizophrenia [15,16]. Changes in these inflammatory markers are directly associated with the disease mechanisms. For instance, it has been hypothesised that inflammation interferes with cellular pathways, inducing the metabolism of tryptophan to kynurenic acid in schizophrenia [17]. The C-reactive protein (CRP) has been shown to correlate with disease severity and is associated with cognitive function in schizophrenia [18,19]. A meta-analysis revealed that serum levels of IL-1RA, IL-6, IL-7, IL-8, IL-9, IL-10, IL-13, IFN-ɣ, eotaxin-1, granulocyte-macrophage colony-stimulating factor, monocyte chemoattractant protein-1 (MCP-1), platelet-derived growth factor subunit B, monocyte inflammatory proteins (MIP-1α and MIP-1β), vascular endothelial growth factor A, and regulated on activation, normal T cell expressed and secreted (RANTES), all associated with the immune/inflammation response and cytokine production, were increased in patients with multi-episode schizophrenia [20,21]. Neurotrophins and their precursors have aroused great interest as possible players in the pathophysiology of several psychiatric disorders, including schizophrenia. Studies have shown significantly reduced brain-derived neurotrophic factor (BDNF), nerve growth factor (NGF), and NGF receptor (NGFR ) levels in the prefrontal cortex (PFC) and the cerebrospinal fluid (CSF) of subjects diagnosed with schizophrenia [22,23]. In accordance with the glutamate–dopamine dysbalance in schizophrenia, imaging studies revealed significant increases in glutamate and glutamine, and reduced dopamine in several brain regions [24,25]. Furthermore, the CSF levels of homovanillic acid, a dopamine metabolite, are decreased in patients with schizophrenia compared with controls [26]. There are no specific biomarkers for the prodrome stage of schizophrenia, however, some markers for the risk and disease progression are proposed (Table 1).

Pharmacological treatments for schizophrenia are limited, and antipsychotics are mainly used in schizophrenia treatment. The 60-year-old dopamine hypothesis remains consistent with the mechanism of action of all currently utilized antipsychotic drugs. Positive symptoms and secondary negative symptoms are generally effectively managed with available antipsychotic treatment, however, antipsychotic medications have little impact on cognitive impairments in schizophrenia, perhaps because the latter is related to different pathophysiological processes. Moreover, the primary negative symptoms generally do not respond well to the currently available antipsychotic treatment with dopamine D_2_ antagonists or partial D_2_ agonists [39]. Unfortunately, some patients (20–30%) show little or no response to the treatment, and even more, patients have treatment-resistant schizophrenia [40,41,42,43,44]. 

Schizophrenia is highly heritable, gene-associated studies identified the risk variants for schizophrenia that aggregate in specific biological pathways (such as postsynaptic density, postsynaptic membrane, dendritic spine, and the axon part of a neuron), but the genetic background does not exclusively explain the aetiology and pathogenesis of schizophrenia. Rather, schizophrenia can be regarded as a polygenic disorder in which genetic variants, when present, contribute small increments of risk. Whereas the original neurodevelopmental model of schizophrenia was based on circumstantial and epidemiological evidence linking the disorder to prenatal and early postnatal life, recent analyses have revealed that early brain development at least partly mediates the genetic risk of schizophrenia, which has fundamental implications for the pathogenesis state [45]. Epigenetic mechanisms, mediating environmental factors to gene expression without directly affecting the DNA sequence, during early brain development are also implicated in schizophrenia risk. Unlike genetic changes, epigenetic changes are reversible but affect gene expression more extensively than genetic changes, which makes them susceptible to pharmacological manipulation.

Thus, despite advances in understanding the aetiology and psychopharmacology of schizophrenia, there is a great need to look for new targets for schizophrenia treatment that might be more effective during therapy.

## 2. Aetiology of Schizophrenia

Schizophrenia is considered a neurodevelopmental disorder with still uncertain aetiology. Genetic and epidemiologic evidence suggests that disease development results from gene–environment interaction affecting neuronal maturation during early life and adolescence [46]. It is also considered that impairments of the epigenetic mechanisms, interacting with environmental risk factors and regulating gene expression, might be involved in the aetiology of schizophrenia [47]. 

### 2.1. Genetic Background

Although genetic studies have failed to identify any DNA variant that contributes to a vulnerability to schizophrenia, several findings indicate a strong genetic background to the disorder. While the incidence of schizophrenia is 1% in the general population, heritability studies have shown that the risk of developing schizophrenia in first- and second-degree relatives is approximately 10% and 3%, respectively, and is further increased for monozygotic twins and dizygotic twins (48% and 14%, respectively). Moreover, the emergence of the condition in the offspring when both parents have schizophrenia is about 40% [48]. 

Genetic findings have shown genomic copy number variations [49], and single nucleotide polymorphisms (SNPs) [50], coupled with alterations in gene expression in patients with schizophrenia [51]. Genome-wide association studies (GWAS) have found more than 100 loci that contain multiple genes associated with schizophrenia [29].

Gene-associated studies have shown a possible relationship between variants of genes related to dopamine signallings, such as catechol O-methyltransferase (COMT), monoamine oxidase (MAO), and dopamine transporter (SLC6A3), and the risk of schizophrenia [52,53]. The allelic variants (Val158Met) in the COMT gene, coding for the dopamine-degrading enzyme, a main regulator of the prefrontal dopamine level, increased the prefrontal dopamine catabolism and impaired prefrontal cognition in schizophrenic patients, while the MAO-B rs 1799736 polymorphism (A to G substitution in the MAO-B enzyme with a role in dopamine degradation and the regulation of dopaminergic neuron activity) was suggested as being connected with the aetiology of schizophrenia and negative symptoms development. Among the genes implicated in schizophrenia, susceptibility variants linked to genes affecting GABAergic transmission (GABA B receptor components GABBR1 and GABBR2, and loci linked to proteins that mediate GABA receptor turnover, such as ankyrin-G (ANK3)) were reported. Genetic advances show that schizophrenia is also associated with excitatory neurotransmission: the N-methyl-D-aspartate (NMDA) receptor (subunit 2A; *GRIN2A*, the estimated odds ratio for highly damaging coding variants ~24.1), the α-amino-3-hydroxy-5-methyl-4-isoxazolepropionic acid (AMPA) receptor subunit (*GRIA3*; the estimated odds ratio for highly damaging coding variants ~20.1), as well as various postsynaptic cell adhesion and scaffolding proteins of excitatory synapses like postsynaptic density protein 93 (PSD-93) and SYNGAP1, which regulate the NMDA receptor-dependent trafficking of AMPA receptors and synaptic potentiation, and are required for proper synaptic function [54]. The abovementioned factors indicate that the genetic predisposition to schizophrenia might be related to the regulation of synaptic neurotransmission (i.e., dopaminergic, glutaminergic, GABAergic) and synaptic plasticity.

Chromosome 22q11. 2 microdeletions provide the most convincing evidence of an association between a molecular cytogenetic abnormality and schizophrenia [55]. One in four individuals born with recurrent 22q11. 2 deletions develop schizophrenia, and ∼0.5–1% of individuals with schizophrenia in the general population have the associated 22q11 [56]. 

However, despite the linkage studies suggesting DNA variants contribute to schizophrenia risk, these findings have not always been replicated and have not yielded results consistent with that estimate of heritability. Some studies determined that the genetic component of schizophrenia may have been overrated, and an increased focus on gene–environmental interactions is likely to accelerate research progress on this disease [57]. Thus, schizophrenia has a complex genetic background with still unclear aetiology.

### 2.2. Environmental Risk Factors

Environmental factors may be extremely complex and the clinical heterogeneity of schizophrenia might be one of several explanations for the discrepancies between genetic studies and the illness outcome. Epidemiological evidence revealed several environmental risk factors that acting at many levels over time, affect brain development and influence the individual’s susceptibility to developing the disorder. The risk factors are possibly acting at crucial stages in brain development encompassing two critical periods: perinatal and adolescence [58]. The risk factors confirmed in several studies and presented before schizophrenia onset include the season of birth, perinatal complications, parental age, infections, and autoimmune diseases [10,59]. It is suggested that environmental risk factors, such as childhood trauma (physical abuse, physical neglect, sexual abuse), and cannabinoids used, especially during adolescence, might be essential triggers for schizophrenia development [60].

### 2.3. Epigenetic Background

The interaction between genetic background and environmental risk factors engages epigenetic mechanisms affecting gene expression without changes in DNA sequences. Several studies provide evidence for the epigenome having a critical function in brain development and disease manifestation [61,62,63]. Epigenomic signatures include DNA methylation, histone variants and modifications, alterations in nucleosome positioning, and non-coding RNAs [63].

#### 2.3.1. Epigenetic Mechanisms

DNA Methylation

DNA methylation is a dynamic process, which is catalyzed by DNA methyltransferase (DNMTs: DNMT1, DNMT3A, DNMT3B, and DNMT3L). DNMT3L has a similarity to other DNMTs, but it does not possess methyltransferase activity. However, it stimulates de novo methylation by the DNA cytosine methyltransferase 3α and is required for the establishment of maternal genomic imprints [64]. DNMTs are involved in the transfer of the methyl group from S-adenyl-L-methionine to the cytosine residues at the C5 position to form 5-methylcytosine (5mC) and homocysteine. Specifically, 5mC is a marker of DNA methylation, whereas the hydroxylated form of 5mC (5hmC: 5-hydroxymethylcytosine, 5-formylcytosine, 5-carboxylcytosine) signifies an active demethylation process initiated by the ten-eleven translocation (TET) family enzyme [65]. DNA methylation at the promoter and enhancer sites regulates the transcriptional activity, mainly repressively [66].

Histone modification

Gene transcription is also regulated by post-translational modifications of histone tails, including methylation, acetylation, phosphorylation, SUMOylation, and ADP-ribosylation, among others. Histone modifications are reversible and involve an antagonistic set of enzymes, such as writers modifying specific substrates by adding groups (enzymes, i.e., histone methyltransferases (HMTs), histone acetyltransferases (HATs), and kinases), and erasers catalysing the removal of specific histone modifications (enzymes, i.e., histone demethylases (HDMs), and histone deacetylases (HDCAs). Specific histone marks are recognized by readers, namely regulatory proteins containing unique domains that recognise specific groups (i.e., methyl, acetyl, and phosphate) [67]. Post-translational histone modifications either promote or repress transcription, and that depends on the kind of modification and the modified amino acid residue. Histone methylation can exist in multiple valence states: mono (me1), di (me2), and trimethylation (me3) forms. The methylation of lysine (K) and arginine (R) residues can be associated with either gene activation or repression depending on the residues being modified, i.e., tri-methylated histone H3 at lysine (K) 4 (H3K4me3) leads to open chromatin, whereas di-methylated histone H3 at lysine (K) 9 (H3K9me2) represses gene expression [67]. Acetylation of several lysine residues throughout the N-terminal tails of core histone proteins is generally correlated with gene activation. Histone modifications together with enzymes using ATP-energy (i.e., Snf2- or SWI/SNF-related enzymes) are involved in the chromatin dynamic regulation [68].

Non-coding RNAs

Non-coding RNAs are functional regulatory RNAs that orchestrate cellular functions and not encoding proteins. They can be divided into small and long non-coding RNAs (sncRNAs and lncRNAs, respectively). LncRNAs (RNAs longer than 200 nucleotides) act on transcription and post-transcriptional events in the nucleus and as a consequence influence translation [69]. SncRNAs mainly act on translation in the cytosol, and to this group belong microRNA (miRNAs), small interfering RNAs (siRNAs), and piwi-interacting RNAs (piRNas). The most studied sncRNA, miRNAs are a family of short (21–25 nucleotides) RNA sequences targeting hundreds of genes via a sequence complementary to the 3′-untranslated region of the mRNA. Subsequently, miRNAs negatively regulate gene expression at the post-transcriptional level. Several findings reveal that one miRNA could regulate the expression of more than one target, and a single mRNA target can be under the control of several miRNAs [70]. 

#### 2.3.2. Human Studies

The role of epigenetic mechanisms acting in humans before the appearance of the first schizophrenia symptom is difficult to investigate because of the trajectory and dynamics of the disease. Some deficits across multiple cognitive domains and reduced social abilities are observed before psychosis begins, but they cannot predict schizophrenia appearance [71]. Studies on children’s neurodevelopmental abnormalities indicate that DNA methylation and histone modifications are essential for normal brain development [72]. Although neurodevelopmental aspects of schizophrenia are difficult to investigate in clinical studies, the involvement of epigenetic regulation in schizophrenia development might be evaluated by genetic and postmortem studies. 

Genetic studies have shown that a high number of schizophrenia risk genes are involved in the epigenetic regulation of gene transcription. These genes are involved in the regulation of histone acetylation, methylation or chromatin remodelling, e.g., B-Cell CLL/lymphoma 11 B (BCL11B B-CE), chromodomain helicase DNA-binding protein 7 (CHD7), histone acetyltransferase E1A-binding protein P300 (P300), enhancer of polycomb homolog 2 (EPC2), lysine-specific demethylase 3B (KDM3B), transcriptional repressor GATAD2A, transcriptional co-repressor RERE, and SATB2 [73]. Moreover, lost-of-function de novo mutations of SETD1A showed the highest statistical significance with neurodevelopmental disorders, i.e., schizophrenia and autism spectrum disorders [74,75,76]. The SETD1A gene encodes trimethyltransferase that governs the presence of H3K4me3 in the genome, and this histone modification might be important for gene expression regulation during brain development [77]. The function of SETDB1, a histone methyltransferase specifically methylating H3K9, and a histone modification involved in transcriptional repression and local heterochromatin formation is also affected in schizophrenia [78]. Moreover, SETDB1 may disrupt the chromatin contacts associated with schizophrenia risk loci [79]. 

Postmortem studies revealed that H3K4me3 tracked GABAergic markers across development, especially the GAD1 gene encoding glutamic acid decarboxylase (GAD) 67, a principle enzyme for GABA synthesis [80,81]. In the schizophrenic brain, a reduction in H3K4me3 levels at GAD1 promoters resulted in deficits in GABA synthesis [81,82]. Postmortem findings also showed a significant hypoacetylation of histone H3 at lysine 9 and 14 (H3K9K14) in the PFC of young subjects with schizophrenia, a phenomenon observed throughout ageing as well [82], suggesting an important role of histone acetylation in schizophrenia development. Concurrently with H3K4me3 reduction, a decrease in H3K9K14ac was observed in the promoter region of the GAD1 gene, and a reduction in GAD1 gene expression was also detected [82]. Notably, GABAergic dysfunctions, especially a decrease in GAD1 expression, are the most replicated findings in the schizophrenia postmortem brain [83], and might be regulated by histone modifications during brain development affecting GABAergic transmission. 

Human postmortem studies have also shown that the expression of miRNA in the brain is age dependent [84] and the adolescent period corresponds to a shift pattern in global miRNA expression [85]. The gene targets of these miRNAs are involved in the development, cell-to-cell signalling and interactions, and they are associated with neurodevelopmental disorders, i.e., schizophrenia [85]. The involvement of miRNAs in schizophrenia is confirmed by several studies. Some findings reported that 16 miRNAs were found to be differentially expressed in the PFC of schizophrenia subjects [86]. Another study also showed that the expression levels of miR-181b, miR-30e, miR-34a, and miR-7, were significantly overexpressed in the plasma of schizophrenia patients [87]. The upregulation of other miRNAs (miR-1273d, miR-1303, miR-21, miR-3064-5, miR-3131, miR-3687, miR-4428, miR-4725-3p, and miR-5096) was also reported in schizophrenia [88].

#### 2.3.3. Animal Studies

Most findings regarding the specific involvement of epigenetic regulation during schizophrenia development are based on results from experimental studies, mainly animal neurodevelopmental models of schizophrenia. There are no animal models that truly mimic schizophrenia, and they have a lot of limitations. As the animal only mimics the behaviour/symptoms, the mechanistic/causative effects of the true disease state can be hard to interpret. However, animal models are suitable to explore the neurodevelopmental aspects of schizophrenia [46].

One of the accepted neurodevelopmental models of schizophrenia is based on the prenatal administration of mitotoxin (methylazoxymethanol acetate (MAM)) on embryonic day 17 (MAM-E17), which induces schizophrenia-like abnormalities in adulthood. The MAM-E17 model has good validity and could model the aetiology, developmental trajectory, and therapy of schizophrenia [89]. At first, epigenetic mechanisms, DNA methylation, was investigated using the MAM-E17 model. The results showed a decrease in DNA methylation at the gene promoter of cannabinoid receptor 1 (CB1) in the adult PFC, which correlated with an increase in the mRNA and protein levels of CB1 at the same age [90]. Moreover, cannabinol treatment during adolescence prevented changes in the CB1 receptor in the PFC of adult MAM-E17 animals and eliminated impairments in social interactions and recognition memory [90]. Recently, methylation patterns and mRNA expression of synapse-relevant genes during adolescence in the MAM-E17 model were analyzed in the gyrus cingulum and the PFC. The changes in the dopamine receptor D2 (Drd2) and dystrobrevin-binding protein 1 (Dtnbp1) gene expression in early adolescence were dependent on the DNA methylation status [91]. Using the MAM-E17 model, total histone methylation during postnatal life was also analyzed in the PFC of rats. Different trajectories of the changes in the H3K9me2 and H3K4me3 proteins were observed. Prenatal MAM administration affected the total H3K9me2 level only in adolescence. In contrast, the global H3K4me3 level was decreased in adulthood [92]. Moreover, a specific reduction in H3K4me3 was found at the promoter of GAD1 and parvalbumin genes that were correlated with a decrease in the mRNA levels of these genes [93,94]. Besides the H3H4me3 protein, an increase in global H3K27me3 was detected in the juvenile PFC of MAM-E17 rats. Additionally, an increase in the levels of H3K27me3 and the transcriptional repressor REST at the proximal promoter region of Grin2b might contribute to the decrease in an NMDA receptor subunit 2B (GluN2B) protein in the early stages of postnatal development in the MAM-E17 model [95]. Another mechanism of epigenetic regulation, histone acetylation, was also studied using the MAM-E17 model. The administration of valproic acid (VPA), a non-selective inhibitor of HDACs, during early adolescence prevented changes in the epigenetic markers, i.e., a decrease in H3K4me3 or an increase in HDAC2 in the adult PFC of MAM-E17 rats [92,96]. Adolescent VPA treatment also abolished deficits in sensorimotor gating in adulthood [96]. Moreover, the pharmacological inhibition of histone acetylation readers, bromodomain and extraterminal (BET) family proteins, during adolescence, impaired cognitive function in adult rats and changed the protein patterns in the adult PFC [97].

The pathogen-free polyinosinic:polycytidylic acid (poly I:C) induced maternal immune activation (MIA), an animal model for neurodevelopmental disorders (schizophrenia, autism spectrum disorder), showed relatively high construct and face validity [98]. Some evidence indicates DNA hypomethylation in the hypothalamus and striatum of adolescent offspring exposed to MIA [99]. Prenatal poly I:C exposure also induced the elevation of H3K9K14 acetylation at the promoter regions genes related to schizophrenia, i.e., Disrupted-in-Schizophrenia 1 (DISC1), nuclear receptor subfamily 2 group F member 1 (Nr2f1), and glutamate ionotropic receptor AMPA type subunits 1 and 2 (Gria1 and Gria2) in the hippocampus of juvenile offspring [100]. Another study using this model revealed global changes in histone acetylation of H3 and H4 as well as at the promoters of a subunit of nuclear factor kappa B (Rela) and genes encoding acetyltransferases, i.e., the CREB-binding protein (CBP) and the E1A-associated protein p300 (EP300) in the PFC of young adult rats prenatally treated with poly I:C. Moreover, an increase in the HDAC6 binding on the promoter region of the Nod-like receptor family pyrin domain containing 3 (Nlrp3) gene was also reported [101]. 

The early-life or adolescent blockade of NMDA receptors induced the schizophrenia-like behavioural response in adult animals [102]. Biochemical studies have shown that behavioural changes are related to the alterations in histone acetylation (H3K9ac) [103] or in HDAC5 levels in the adult PFC of animals with postnatal blockade of NMDA receptors [103,104]. 

In the animal model of mice dams stressed during pregnancy, the offspring of mothers subjected to registrant stress during pregnancy are vulnerable to developing schizophrenia-like behavioural abnormalities, i.e., deficits in social interaction, sensorimotor gating or fear conditioning. Moreover, changes in the DNA methylation/demethylation processes were analyzed in the offspring at birth and postnatal days 7, 14 and 21. The levels of DNMT1 mRNA and Tet methylcytosine dioxygenase 1 (TET1) were higher in the offspring of prenatally stressed mice than in the control animals [105].

The above findings indicate that DNA methylation and histone modifications, mainly methylation and acetylation, appear to be involved in abnormal brain maturation and schizophrenia development (Table 2).

## 3. Epigenetic Regulation as Therapeutic Targets

Epigenetic modifications are dynamic and reversible and epigenetically provoked alterations of brain development can be evaded by using specific molecules acting to improve epigenetic malfunctions. These drugs might restore or reverse gene expression abnormalities contributing to disease manifestation.

### 3.1. DNA Methylation

Some evidence indicates that DNMT inhibitors might regulate the expression of some genes related to schizophrenia (Reelin (RELN), GAD1). Specifically, Aza-2′-deoxycytidine, a nonmethylatable cytosine analogue that is an inhibitor of DNMT1, reduced the intensity of methylation at the reelin promoter region and increased the expression of reelin in cultured neural progenitor cells [106]. Human RELN and GAD1 gene expression in neuronal precursor cells were also improved by DNMT1 inhibitors, doxorubicin and zebularine [107]. Recently, a DNMT1 inhibitor, N-phthalyl-l-tryptophan (RG108) [108], showed positive effects on altered behavioural (deficits in social behaviour) and molecular (hypermethylation of GAD67, RELN, BDNF genes) endophenotypes in prenatally stressed mice (PRS neurodevelopmental model) [109]. DNMT inhibitors are clinically used for cancer treatment [110,111], but currently, they are not considered in schizophrenia therapy, partially because of the low blood–brain barrier permeability [112]. 

### 3.2. Histone Modifications

#### 3.2.1. Histone Acetylation

HDAC inhibitors have gained considerable attention as a relevant therapeutic option for psychiatric disorders. In humans, HDAC enzymes are grouped into four classes: the class I Rpd3-like protein (HDAC1, HDAC2, HDAC3, and HDAC8); the class II Hda1-like protein (HDAC4, HDAC5, HDAC6, HDAC7, HDAC9, and HDAC10); the class III Sir2 -like proteins (SIRT1, SIRT2, SIRT3, SIRT4, SIRT5, SIRT 6, and SIRT 7); and the class IV protein (HDAC11) [113,114].

VPA is, among other actions, a non-selective inhibitor of class I and II HDACs [115]. Animal studies have shown the positive effects of VPA on schizophrenia-like abnormalities [96,105]. In clinical practice, VPA treatment showed beneficial effects in schizophrenia patients [116], and VPA is used in augmentation therapy for treatment-resistant schizophrenia [117]. Other HDACs of class I and II inhibitors (MS-275, vorinostat, sodium butyrate) also showed potential beneficial effects on schizophrenia-like impairments in animal studies [104,118,119,120]. There are also some suggestions that HDAC10 might be, among others, a potential target for schizophrenia [121] because of a single nucleotide polymorphism (SNP) (rs 7634112) located in the HDAC10 gene associated with schizophrenia [122]. Thus, HDAC inhibitors might be a promising compound in schizophrenia treatment, especially in the potential therapy of schizophrenia-resistant patients, however, they are still being checked in clinical trials.

Recently, the inhibition of histone acetylation readers from the BET family protein is deliberated to have potential in schizophrenia therapy since the BET family inhibitor, JQ1 improves transcriptional abnormalities in the neurones of schizophrenia patients [123]. On the other hand, in animal studies, the pharmacological inhibition of the BET family protein during adolescence, induced schizophrenia-like abnormalities in adulthood [97]. Thus, the BET family might be an important target for schizophrenia development and treatment, however, there is a need for further studies of the exact role of BET proteins in these processes.

#### 3.2.2. Histone Methylation

Some evidence indicates that HTMs might be a potential target in schizophrenia treatment [124,125]. HTMs are divided into lysine methyltransferases (KMTs) and arginine methyltransferases (PRMTs). KMTs are grouped into SET domain-containing and non-SET domain-containing proteins. SET methyltransferases are subdivided into different families, i.e., SET1 (EZH1, EZH2), and SUV 39 (SUV39H1, SUV39H2, SETDB1, G9a, GLP) [126]. 

BIX-01294, an inhibitor of G9a methyltransferase reduced global methylation and promoter-specific H3K9me2 levels in the peripheral blood cells of patients with schizophrenia affects the expression of some genes (IL-6, NANOG, GAD67, KLF4) [127]. 

Animal studies have shown that impairments in methyltransferase function induced schizophrenia-like phenotypes. EZH1 methyltransferase dysregulation changed the response to antipsychotic treatment and altered sociability and motivation behaviour [128]. On the other hand, a SETD1A dysfunction impaired sociality and motivation for social interaction [129], and a SETDB1 malfunction impaired working memory [130]. The abovementioned data confirmed the observation that molecules targeting methyltransferases might be effective in schizophrenia therapy. 

## 4. Effects of Antipsychotics on Epigenetic Dysfunction in Schizophrenia

The available pharmacotherapeutic options for schizophrenia treatment are symptomatic and apply after psychotic onset. There are two main classes of antipsychotics: typical and atypical. Typical antipsychotics are primarily dopamine D2 antagonists (haloperidol, chlorpromazine, fluphenazine). Atypical antipsychotics also have affinities other than D2 receptors (clozapine, risperidone, quetiapine, olanzapine). Current antipsychotics are targeted mainly at psychotic symptoms with limited effect on the negative and cognitive impairments. There are also several side effects related to antipsychotic treatment, i.e., extrapyramidal, and cardio-metabolic dysfunction. Moreover, more than 30% of patients are resistant to pharmacotherapy [42]. Thus, the dopamine D2 receptor blockade cannot explain all aspects of the therapeutic effects of antipsychotics. Therefore, several findings indicate that some therapeutic effects of antipsychotics might be related to their influences on epigenetic modifications. Moreover, it is suggested that epigenetic alterations in schizophrenia might affect the treatment response in patients, especially those resistant to pharmacotherapy [131].

### 4.1. Human Studies

A functional polymorphism, rs6295 in the 5-HT1A receptor gene (HTR1A), is known to be associated with the negative schizophrenia symptom response to antipsychotic medication. Analysis of DNA methylation at a specific CpG site adjacent to the functional polymorphism rs6295 in schizophrenia patients has shown a correlation between the change in negative symptoms following the initial antipsychotic treatment (clozapine, chlorpromazine, risperidone, fluphenazine) with methylation determined before the onset of treatment. The result suggests that epigenetic variation and genetic polymorphism in a specific DNA sequence can affect negative symptoms and responses to antipsychotics [132]. DNA hypermethylation of the DTNBP1 promoter gene was also reported in schizophrenia patients. Antipsychotic treatment, particularly classic antipsychotic drugs, induced a decrease in DNA methylation and induced the expression of DTNBP1 [133] DNA methylation of the ankyrin repeat and kinase domain containing 1 (ANKK1) in the response to an antipsychotic, aripiprazole, was investigated in acute schizophrenia patients. The results showed that DNA methylation levels at CpG site 387 of ANKK1 were higher in the aripiprazole-treated group and correlated with the changes in the positive and negative syndrome scale scores (PANSS). Moreover, responders’ methylation of all CpG sites was significantly correlated with the plasma levels of monoamine metabolites (homovanillic acid (HVA), and 3-methoxy-4hydroxyphenylglycol (MHPG)). The ANKK1 CpG site 387 is a target of the CCCTC-binding factor that might potentially alter the dopamine D2 density and the methylation of ANKK1 might affect dopaminergic transmission in schizophrenia treatment [134]. Risperidone treatment altered the DNA methylation in 5979 CpG sites in first-episode schizophrenia patients. Moreover, the DNA methylation changes normalized after clozapine therapy were correlated with the symptom severity (PANSS) and cognitive function (Stroop Color Word Test (SCWT), Wisconsin Card Sorting Test (WCST), Trail Making Test (TMT), Verbal Fluency Test (VFT), Digit Span Distraction Test (DSDT)) [135]. Antipsychotic treatment (risperidone, olanzapine, aripiprazole) also reversed hypomethylation in the interleukin 6 (IL-6) gene promoter in schizophrenia subjects [136]. 

Another study identified the genes associated with the antipsychotic treatment response in schizophrenia patients (DNMT3A rs2304429, HDAC5 rs11079983, HDAC9 rs1178119) [137]. The abovementioned genes encode the proteins involved in the regulation of epigenetic modifications. Apart from DNA methylation, histone modifications might be also involved in the effects of antipsychotics on gene expression in schizophrenia. The upregulation of ADRA2A expression (encoding α2A-adrenoreceptors) in antipsychotic-treated schizophrenia subjects was related to changes in the histone methylation (increased H3K4me3 and H3K27me3) and acetylation (enhanced H4K16ac) levels at the ADRA2A promoter [138]. Several findings indicate that antipsychotics affect miRNA levels. Antipsychotic treatment (olanzapine, quetiapine, ziprasidone, risperidone) significantly downregulated miR-181b, and that effect was positively correlated with the improvement of negative symptoms [87]. However, a decrease in expression in miR-21 after treatment (olanzapine, quetiapine, ziprasidone, risperidone) was negatively correlated with the improvement of positive, general psychopathology, and aggressiveness symptoms [88].

Clozapine is the only FDA-approved antipsychotic for treatment-resistant schizophrenia patients [40,42]. The effects of clozapine on DNA methylation in treatment-resistant schizophrenia patients were investigated using peripheral blood samples. The DNA methylation changes following clozapine treatment were enriched in the genes related to cell-substrate adhesion and cell-matrix adhesion. Clozapine treatment induced significant CpG sites of the decreased DNA methylation, which were located in CpG islands in the promoter regions of the genes related to GABA (GAD1) and glutamate (glutamate ionotropic receptor NMDA type subunit 2A (GRIN2A), glutamate ionotropic receptor NMDA type subunit 2D (GRIN2D), glutamate metabotropic receptor 7 (GRM7)). The increase in DNA methylation of the CREB-binding proteins (CREBBP) gene induced by clozapine was significantly correlated with the clinical improvements (PANSS) in treatment-resistant schizophrenia [139]. Methylation analysis in monozygotic twins with treatment-resistant schizophrenia, in which one responded to clozapine treatment, and the second did not, was also performed. Genome-wide DNA methylation and transcriptome (RNA-seq. data) profiling revealed the different proportions of altered methylation and expression of genes related to the neuronal and synaptic function between the clozapine responder and non-responder (35.7 vs. 6.7%, respectively) [140]. Another study revealed that some miRNAs (miR-181b, miR-195-5p, and miR-195-5p) expressions were different between the treatment-resistant group and the group responding well to antipsychotic therapy [141].

The available findings suggest that antipsychotic therapy affected the epigenetic status of genes related to schizophrenia (Table 3).

### 4.2. Animal Studies

Clozapine and sulpiride, but not haloperidol and olanzapine, increased the cortical and striatal demethylation of the hypermethylated RELN and GAD 1 promotor in mice pretreated with l-methionine [142]. Olanzapine caused DNA methylation changes in the genes encoding dopamine receptors (D1, D2, D5), dopamine transporter (solute carrier family 18 members 2 (SLC18A2)), and dopamine metabolism (COMT) in the rat brain [143]. 

Clozapine, but not haloperidol or risperidone, corrects behavioural phenotypes and normalized the DNMT1 level and 5-mC promoter hypermethylation of schizophrenia-related genes (GAD1, RELN, BDNF) in the PRS animal models [109]. 

The animal models based on the blockade of the NMDA receptors by antagonists (phencyclidine, MK-801, ketamine) also analyzed the effect of antipsychotics on behavioural responses and epigenetic alterations. Clozapine, but not haloperidol, attenuated the deficits in memory and social interactions induced by repeated phencyclidine administration. Moreover, clozapine normalized the increase in HDAC5 levels and the decrease in H3K9ac in the PFC. The abovementioned effects were related to the D1 receptor activation in the PFC [144]. Another atypical neuroleptic, risperidone, normalized an increase in histone H3 phosphorylation (H3S10p) in the PFC induced by a single injection of MK-801 [145]. Another finding showed that acute phencyclidine administration reduced miR-143 in plasma, the PFC and the hippocampus of mice. The antipsychotics, haloperidol and clozapine, attenuated hyperlocomotion and a decrease in miRNA-143 expression, and the abovementioned effects were mediated by the dopamine D2 receptor [146].

Haloperidol administration increased the expression of miR-199a, miR-128a, and miR-128b in the rat cortex [87]. Another study investigated the effect of antipsychotic treatment (clozapine, olanzapine or haloperidol) on miRNAs expression in mice brains, which revealed that clozapine upregulated five miRNAs (miR-342-5p, miR-1198, miR-31, miR-329, miR-337-5p), olanzapine downregulated five miRNAs (miR-690, miR-193, miR-218-2, miR-223, miR-544), and haloperidol also downregulated six miRNAs (miR-378, miR-22, miR-218-2, miR-339-5p, miR-434 -5p, miR-410) [147].

## 5. Discussion

The present study reviewed the available data related to epigenetic regulation in schizophrenia based on peer-reviewed studies published in English by international journals. Investigations of the epigenetic mechanisms in schizophrenia pathomechanism started more than 10 years ago, but there are still a lot of questions related to schizophrenia aetiology, development, and treatment. 

The role of epigenetic modifications in proper neurodevelopment and their impairments in schizophrenia are mainly based on animal schizophrenia models and limited human studies. The available evidence mainly concentrates on the epigenetic mechanisms related to DNA methylation, histone modifications or miRNA expression. Several findings suggest that epigenetic regulation is dynamic and reversible. Thus, several factors, mainly environmental, despite genetic background, might prevent schizophrenia development or accelerate symptom appearance. The precise and early diagnosis of schizophrenia is challenging, and therapy before the first psychotic episode is not offered because there are no specific biomarkers or signs in front of the initial psychotic episode of schizophrenia. A better understanding of the trajectory of schizophrenia development might be helpful for the prevention of schizophrenia’s appearance, using for example enrichment environments during childhood and adolescence. The present knowledge about biochemical and molecular changes during brain maturation is mainly based on animal models that do not exactly correlate with human development. Epidemiological studies showing data from different human populations might support recognizing the prodromal stage of schizophrenia. Another advantage would be to find biomarkers specific to the early stages of developing the disease. However, such studies are difficult to process because of the complexity of schizophrenia aetiology. Heterogenous genetic background and environmental impact might be the reasons for the failure in defining schizophrenia markers before their first appearance, even in families with a schizophrenia history.

Epigenetic drugs targeting epigenetic regulators are still under investigation in schizophrenia treatment. Although preclinical studies revealed that molecules acting through epigenetic mechanisms, i.e., DNA methyltransferases or HDAC inhibitors, might have therapeutic potential, they are not used in schizophrenia therapy (DNA methyltransferase) or they are subject to clinical trials (HDAC inhibitors). Regulation of histone acetylation appears to be essential for schizophrenia development and therapy in the context of the recent data related to the acetylation readers from the BET family. The results from animal studies have shown the development of schizophrenia-like abnormalities induced by the inhibition of BET proteins in adolescence. On the other hand, the beneficial effect of BET protein inhibitors was found in abnormalities in schizophrenia patients. The above findings might suggest that the age (adolescence vs. adulthood) and the stage of schizophrenia development are important for the therapeutic effectiveness of epigenetic drugs. Moreover, it should be taken into account that histone modification readers might be more effective in potential therapy than other histone regulators, such as writers or erasers. The available epigenetic drugs also have some limitations, i.e., low blood–brain barrier permeability. Moreover, epigenetic modifications do not function alone but cooperate in various combinations. Thus, the prediction of the functional effects of epigenome modifiers may be challenging. However, there is a great need to search for and develop new molecules acting through epigenetic targets. The design of new compounds should have good bioavailability and be verified through animal models of schizophrenia before clinical trials.

Several findings have shown that antipsychotics used in schizophrenia therapy affected epigenetic regulation, which is impaired in schizophrenia, and changed abnormal gene expression related to dopamine, serotonin or glutamate transmission. At present, it is difficult to determine which epigenetic regulations are essential for antipsychotic effects. Future studies using ChiP-seq., RNA-seq., and gene-wide DNA methylation techniques might provide additional information about the impact of antipsychotics on specific epigenetic regulation of the expression of particular genes. The abovementioned results should be correlated with the specific symptoms of schizophrenia. Such an approach might be particularly important for patients with treatment-resistant schizophrenia. Although there are several hypotheses about the neurobiological background of resistance to antipsychotic therapy, there is still only one antipsychotic, clozapine, that is effective in this case of schizophrenia. Currently, there is a limited number of articles related to the epigenetic regulation involved in clozapine therapeutic action in treatment-resistant schizophrenia. However, some of them suggest that combination therapy using clozapine and VPA might be beneficial for the patients. This observation might support the idea of the involvement of epigenetic mechanisms in developing treatment-resistant schizophrenia and indicates future strategies for more successful therapy. Thus, more studies are needed to understand the effects of antipsychotics on epigenetic regulation, as well as the contribution of epigenetic mechanisms to specific symptoms of schizophrenia. 

## 6. Conclusions

The role of epigenetic mechanisms in schizophrenia development is still under investigation. Because of schizophrenia’s trajectory of development, it is difficult to study the background of the disease before the symptoms appeared. Thus, pharmacotherapy in early schizophrenia is challenging and the identification of the risks of schizophrenia would benefit from early diagnosis and pharmacotherapeutic interventions. Several findings, mainly from animal models, suggest a strong epigenetic background to schizophrenia emergence. It might be predicted that epigenetic modifications made them a good target for schizophrenia treatment, however, due to complex interplay, the implications of using epigenetic drugs need more investigation. The link between epigenetic alterations and antipsychotic treatment outcomes is at the beginning of an understanding. Consequently, potential antipsychotic-induced changes in the epigenetic regulation of the expression of schizophrenia risk genes might be essential in treatment-resistant schizophrenia. 

The study has some limitations. First of all, the specific inclusion/exclusion criteria were not used in this review. The online databases (PubMed, Scopus) were used for the literature search, however, the studies presented in the review might not cover all the available data. A publication time limit was not specified, however, the majority of studies were published within the last five years to capture the most recent work. A few articles were published more than ten years ago, but they included some important information related to epigenetics and schizophrenia. Thus, the present review is a compilation of previous and more recent evidence related to epigenetics and schizophrenia. Despite the presented data supporting the role of epigenetics in schizophrenia pathomechanisms, there are still some neurodevelopmental phases not completely understood that need to be addressed in future studies, i.e., which developmental stage is the most important in schizophrenia development from an epigenetic point of view. The available data also do not provide sufficient information related to the role of epigenetic regulation in antipsychotic action. 

Despite the abovementioned limitations, the review presents contemporary knowledge about the epigenetic background of schizophrenia. Theoretically, epigenetic regulations are a promising target for a new therapy for schizophrenia because of their ability to change the expression of genes impaired by the disease and induce schizophrenia recovery. However, there are still some gaps in the knowledge of epigenetic mechanisms that are a result of limited human data and partial translational applications of the result from animal models. The increasing number of human studies analyzing the role of epigenetic regulations on the effectiveness of antipsychotics on defined schizophrenia symptoms might result in further progress in the development of therapy based on epigenetic targets.

## Figures and Tables

**Table 1:** Diagnostic and prognostic biomarkers for schizophrenia.

Regulation	Biomarker	Structure	Diagnostic	References
Peripheral and metabolic	NGF, BDNF, Hcy VitB_6_	serum blood serum serum serum	disorder	[22,23] [27] [28]
Genetic	22q11. 2 MHC regions, MIR137 ZNF804A, NRGN (TCF4)	CNV GWAS	*risk*	[14,29]
Inflammatory	CRP CRP IL-1RA, IL-6, IL-7, IL-8, IL-9, IL-10, IL-13, IFN-ɣ, eotaxin-1, MCP-1, PDGFB, MIP-1α and MIP-1β, VEGF, CCL5 IL-1β, IL-12, TNF-α, sIL-2R cytokine imbalance of T helper types 1 and 2, IL-6 and IL-8 IL-1α, IFN-ɣ-inducible protein 10, IFN-α	serum serum serum CSF CSF brain	disorder	[18,19,20,30,31] [32,33,34]
Neurotransmitter	dopamine glutamate norepinephrine	brain serum urine	disorder	[24,35,36,37,38]

BDNF: brain-derived neurotrophic factor; CNV: copy number variant; CRP: C-reactive protein; CSF: cerebrospinal fluid; GWAS: genome-wide association studies; Hcy: homocysteine; IFN: interferon; IL: interleukin; NGF: nerve growth factor; sIL-2R: soluble IL-2 receptor; TNF: tumour necrosis factor; VitB: vitamin B.

**Table 2:** Epigenetic regulation of gene expression in animal models of schizophrenia.

Animal Model	Age	Epigenetic Regulation	Gene	Structure	References
MAM-E17 model	juvenile	Histone methylation (H3K27me3)	Grin2b	PFC	[95]
	adolescence	DNA methylation	Drd2, Dtnbp1	PFC,	[91]
	adulthood	DNA methylation Histone methylation (H3K4me3)	CNR1 Gad1, PV	PFC, PFC	[90] [93,94]
MIA model	juvenile	Histone acetylation (H3K9K14)	Disc1, Nr2f1, Gria1, Gria2	Hp	[100]
	adulthood	Global histone acetylation (H3 and H4) HDAC6	Rela, CBP, EP300 Nlrp3	PFC	[101]

PFC: prefrontal cortex; Hp: hippocampus; PV: parvalbumin; CNR1: a gene encoding CB1 receptor; Dtnbp1: dystrobrevin-binding protein 1; Drd2: dopamine receptor 2; Grin2b: an NMDA receptor subunit 2B (GluN2B); Disc1: Disrupted-in-Schizophrenia 1; Nr2f1: nuclear receptor subfamily 2 group F member 1; Gria1: glutamate ionotropic receptor AMPA subunit 1; Gria2: glutamate ionotropic receptor AMPA subunit 2; Rela: a subunit of nuclear factor kappa B; CBP: CREB-binding protein; EP300: E1A-associated protein p300; Nlrp3: Nod-like receptor family pyrin domain containing 3.

**Table 3:** Effects of antipsychotics on epigenetic regulation of gene expression in patients with schizophrenia.

Antipsychotics	Epigenetic Regulation	Genes	References
Clozapine, chlorpromazine, risperidone, fluphenazine	DNA methylation	HTR1A (rs6295)	[132]
Classic antipsychotics	DNA methylation	DTNBP1	[133]
Aripiprazole	DNA methylation	ANKK1	[134]
Risperidone, olanzapine, aripiprazole	DNA methylation	IL-6	[136]
Clotiapine, levomepromazine clozapine, aminosulpride, olanzapine, risperidone, sulpiride, quetiapine	Histone acetylation (H4K16ac) Histone methylation (H3K4 me3)	ADRA2A	[138]
Clozapine	DNA methylation	GAD1, GRIN2A, GRIN2D GRM7 CREBBP	[139]

HTR1A: 5-HT1A serotonin receptor; DTNBP1: dystrobrevin-binding protein 1; ANKK1: ankyrin repeat and kinase domain containing 1; IL-6: interleukin 6; ADRA2A: α2A adrenoreceptor; GRIN2A: glutamate ionotropic receptor NMDA type subunit 2A; GRIN2D: glutamate ionotropic receptor NMDA type subunit 2D; GRM7: glutamate metabotropic receptor 7; CREBBP: CREB-binding proteins.

## Data Availability

Not applicable.
